# Association between cumulative glucocorticoid exposure and epicardial adipose tissue volume in systemic lupus erythematosus

**DOI:** 10.1016/j.ero.2025.12.009

**Published:** 2026-01-12

**Authors:** Lévi-Dan Azoulay, Nadjia Kachenoura, Samia Boussouar, Etienne Charpentier, Nassim Ait-Abdallah, Micheline Pha, Miguel Hié, Alexis Mathian, Fleur Cohen-Aubart, Julien Haroche, Zahir Amoura, Alban Redheuil

**Affiliations:** Sorbonne Université, Laboratoire d'Imagerie Biomédicale, Inserm, CNRS, Paris, France; Assistance Publique-Hôpitaux de Paris, Service de Médecine Interne 2, Centre National de Référence du Lupus, Hôpital Pitié-Salpêtrière, Paris, France; Sorbonne Université, Laboratoire d'Imagerie Biomédicale, Inserm, CNRS, Paris, France; Sorbonne Université, Assistance Publique-Hôpitaux de Paris, Unité d’Imagerie Cardiovasculaire et Thoracique (ICT), Institut de Cardiologie, Hôpital Pitié-Salpêtrière, 47-83 Boulevard de l’hôpital, Paris, France; Sorbonne Université, Assistance Publique-Hôpitaux de Paris, Unité d’Imagerie Cardiovasculaire et Thoracique (ICT), Institut de Cardiologie, Hôpital Pitié-Salpêtrière, 47-83 Boulevard de l’hôpital, Paris, France; Sorbonne Université, Assistance Publique-Hôpitaux de Paris, Service de Médecine Interne 2, Centre National de Référence du Lupus, Hôpital Pitié-Salpêtrière, 47-83 Boulevard de l’hôpital, Paris, France; Sorbonne Université, Assistance Publique-Hôpitaux de Paris, Service de Médecine Interne 2, Centre National de Référence du Lupus, Hôpital Pitié-Salpêtrière, 47-83 Boulevard de l’hôpital, Paris, France; Sorbonne Université, Assistance Publique-Hôpitaux de Paris, Service de Médecine Interne 2, Centre National de Référence du Lupus, Hôpital Pitié-Salpêtrière, 47-83 Boulevard de l’hôpital, Paris, France; Sorbonne Université, Assistance Publique-Hôpitaux de Paris, Service de Médecine Interne 2, Centre National de Référence du Lupus, Hôpital Pitié-Salpêtrière, 47-83 Boulevard de l’hôpital, Paris, France; Sorbonne Université, Assistance Publique-Hôpitaux de Paris, Service de Médecine Interne 2, Centre National de Référence du Lupus, Hôpital Pitié-Salpêtrière, 47-83 Boulevard de l’hôpital, Paris, France; Sorbonne Université, Assistance Publique-Hôpitaux de Paris, Service de Médecine Interne 2, Centre National de Référence du Lupus, Hôpital Pitié-Salpêtrière, 47-83 Boulevard de l’hôpital, Paris, France; Sorbonne Université, Assistance Publique-Hôpitaux de Paris, Service de Médecine Interne 2, Centre National de Référence du Lupus, Hôpital Pitié-Salpêtrière, 47-83 Boulevard de l’hôpital, Paris, France; Sorbonne Université, Laboratoire d'Imagerie Biomédicale, Inserm, CNRS, Paris, France; Sorbonne Université, Assistance Publique-Hôpitaux de Paris, Unité d’Imagerie Cardiovasculaire et Thoracique (ICT), Institut de Cardiologie, Hôpital Pitié-Salpêtrière, 47-83 Boulevard de l’hôpital, Paris, France; Sorbonne Université, Assistance Publique-Hôpitaux de Paris, Institut de Cardiométabolisme et de Nutrition (ICAN), Paris, France

Dear Editor,

During the past decade, epicardial adipose tissue (EAT) volume has emerged as a promising cardiovascular risk biomarker both in the general population and in systemic lupus erythematosus (SLE) [1,2]. In 2012, Lipson et al [3] reported that patients with SLE had increased EAT volumes when compared with matched controls. In their study, EAT volume was independently associated with triglycerides and cumulative glucocorticoid (GC) exposure, although disease activity and damage indices were not considered [3]. Therefore, it remains unclear whether EAT volume is increased by disease activity or GC exposure.

Here, we aimed at identifying factors independently associated with EAT volume in SLE.

This retrospective longitudinal study was approved by an institutional review board (#*CER-2024-PROMISE-00322*). Written consent was waived in accordance with the French Law. Data from this cohort have been previously described [2].

Consecutive patients with SLE fulfilling the American College of Rheumatology/European Alliance of Associations for Rheumatology (ACR/EULAR) 2019 criteria who underwent a noncontrast cardiac computed tomography (CT) imaging between January 2014 and January 2024 were retrospectively included. A longstanding systematic coronary artery calcium (CAC) screening policy enabled the establishment of a cohort of unselected primary prevention patients who underwent comprehensive cardiovascular assessment, including cardiac CT. GC cumulative exposure was assessed for each patient and categorised according to the median value (ie, 15 g). EAT volume (mL) was measured through a fully automated artificial intelligence-based segmentation of the cardiac area with a thresholding centred on the density range of adipose tissue values (−190 to −30 Hounsfield units) and normalised over the body surface area (indexed EAT [EATi])*.* CT examinations were analysed using *syngo*.via (Siemens Healthineers) by a single operator (L-D.A) blinded to the patient’s case status.

Statistical analyses were performed using R software, version 4.2.3 (R Project for Statistical Computing). Univariable and multivariable linear regression analyses were performed. Variables included in the analyses encompassed traditional risk factors, SLE disease activity and damage indices, and GC exposure. Variables were included in the multivariable model provided they had a *P* value ≤.1 on univariable analysis. The natural logarithm of EATi volume was used to fulfil the normality assumption.

A total of 231 patients were included (45 ± 13 years, 90% women). Mean SLE duration was 13 ± 9 years. Mean SLE damage index was 0.4 ± 0.9. Seventy-six patients (34%) had a prior history of lupus nephritis, 154 (67%) were on GC, and 116 (50%) had received a least one disease-modifying antirheumatic drug. Cardiovascular risk factors were as follows: hypertension 26%, current smoker 15%, diabetes 6%, body mass index (BMI) 25 ± 6 kg/m^2^, low-density lipoprotein levels 1 ± 0.4 g/L, triglycerides 0.9 ± 0.5 g/L, and estimated glomerular filtration rate (eGFR) 92 ± 21 mL/min/1.73m^2^. Eleven patients (5%) were on lipid-lowering therapy. Mean EATi volume was 63 ± 34 mL/m^2^.

On univariable analysis, age, male sex, diabetes, triglycerides, BMI, eGFR, smoking, lipid-lowering therapy, disease-modifying antirheumatic drugs, SLE damage index, and increased GC exposure were significantly associated with EATi volume (Table). On multivariable analysis, age, male sex, BMI, smoking, and increased GC exposure remained significantly and independently associated with EATi volume (Table).

**Table:** Multivariable regression analysis of epicardial adipose tissue volume in systemic lupus erythematosus

	Univariate analysis		Multivariable analysis^a^	
	β	*P* value	β	*P* value
EATi volume^b^				
Cardiovascular risk				
Age > 50 y	**0.5**	**<.001**	**0.4**	**<.001**
Male sex	**0.5**	**<.001**	**0.4**	**<.001**
Diabetes mellitus	**0.3**	**.04**	−0.1	.4
Triglycerides	**0.3**	**<.001**	0.2	**.02**
Body mass index	**0.04**	**<.001**	**0.03**	**<.001**
eGFR using CKD-EPI	−**0.01**	**<.001**	0	.7
Smoking	**0.3**	**<.001**	**0.2**	**<.001**
Lipid-lowering therapy	**0.3**	**.03**	−0.02	.9
Systemic lupus erythematosus				
Lupus nephritis	−0.01	.9	-	-
SLEDAI	−0.01	.6	-	-
SDI	**0.1**	**<.001**	0.005	.9
DMARDs	**0.09**	**.07**	0.01	.8
Glucocorticoid exposure				
GC exposure >15 g^c^	**0.3**	**<.001**	**0.1**	**.03**

CV, cardiovascular; DMARDs, disease-modifying antirheumatic drugs; EATi, indexed epicardial adipose tissue; eGFR, estimated glomerular filtration rate; GC, glucocorticoid; SDI, SLE damage index; SLE, systemic lupus erythematosus; SLEDAI, SLE disease activity index.

Significant *P* values are bolded. Variables were included in the multivariable model provided their *P* value was ≤.1 on univariate analysis.

a Adjusted R-squared: 0.45; the assumptions of linear regression, including linearity, independence of errors, homoscedasticity, and normally distributed residuals, were all satisfied.

b The natural logarithm of EATi volume was computed to fulfil the normality assumption of linear regression.

c GC cumulative exposure was categorised according to the median value (ie, 15 g).

Overall, high cumulative GC exposure was independently and positively associated with EAT volume. Independent associations were also observed with age, male sex, triglycerides, BMI, and smoking, consistent with Lipson et al [3].

EAT is a modifiable risk factor itself that exerts direct atherogenic effects *per se* and directly contributes to coronary plaques [1]. Altogether, these data could perhaps suggest that EAT may mediate the adverse cardiovascular effects of GC in SLE. Despite limitations inherent to its retrospective and single-centre design, which include selection and documentation biases, the study findings support GC-sparing regimens and pave the way for using GLP-1 receptor agonists and SGLT2 inhibitors to target EAT in SLE [1].

## Competing interests

ZA reports a relationship with GSK, AstraZeneca, Roche, Novartis, Amgen, and Kezar that includes consulting or advisory and funding grants. All other authors declare that they have no known competing financial interests or personal relationships that could have appeared to influence the work reported in this paper. This work was performed without any relationship to the industry.

## References

[1] Iacobellis G. (2022). Epicardial adipose tissue in contemporary cardiology. Nat Rev Cardiol.

[2] Azoulay L-D, Kachenoura N., Boussouar S. (2025). Prognostic value of CT-derived epicardial adipose tissue volume over coronary artery calcium in systemic lupus erythematosus. Eur J Prev Cardiol.

[3] Lipson A., Alexopoulos N., Hartlage G.R. (2012). Epicardial adipose tissue is increased in patients with systemic lupus erythematosus. Atherosclerosis.

